# A long-term survivor with esophageal melanoma and pulmonary metastasis after single-stage esophagectomy and lobectomy: Case report and literature review

**DOI:** 10.1097/MD.0000000000007003

**Published:** 2017-05-26

**Authors:** Tian Zhao, Feng-Wei Kong, Heng Wang, Dong Liu, Chun-Ying Wang, Jin-Hua Luo, Miao Zhang, Wen-Bin Wu

**Affiliations:** a Department of Thoracic Surgery, Xuzhou Central Hospital Affiliated to Southeast University; b Department of General Surgery, Xuzhou Infectious Disease Hospital, Xuzhou; c Department of Thoracic Surgery, Jiangsu Province Hospital, the First Affiliated Hospital of Nanjing Medical University, Nanjing, China.

**Keywords:** interferon, noncutaneous melanoma, primary malignant melanoma of the esophagus (PMME)

## Abstract

**Rationale:**

The optimal therapeutic regimen for primary malignant melanoma of the esophagus (PMME) need to be further elucidated. Besides, the efficacy of surgery for PMME with remote metastasis is uncertain for its rarity.

**Patient concerns:**

Herein a previously healthy patient was admitted for dysphagia and fatigue, without significant weight loss.

**Diagnoses:**

The pathological and molecular tests revealed his diagnosis of BRAF-mutant, advanced PMME with localized pulmonary metastasis.

**Interventions:**

Single-stage Ivor-Lewis esophagectomy and lobectomy were performed successfully, followed by 4 cycles of conventional chemotherapy, and concurrent high-dose interferon lasting for 1 year.

**Outcomes:**

The patient survived without logo-regional recurrence or remote metastasis during the follow up of two and a half years up to now.

**Lessons:**

Timely resection of localized primary and metastatic lesions might deliver a chance to obtain better prognosis for selected PMME patients; however, high-quality trials with longer follow-up are needed.

## 1 Introduction

Primary malignant melanoma of the esophagus (PMME) is rare but highly aggressive. It was firstly described in 1964, and represents only nearly 0.1% of all malignant esophageal neoplasms, with a poor prognosis.^[1]^ Besides, the patients are usually diagnosed at a late stage because the manifestations are mainly nonspecific. The most common metastasis organs form PMME are liver, mediastinum, lung, and brain.^[2]^

However, comprehensive understanding of PMME is hard for the rarity of this disease; consequently, the optimal therapeutic strategy including aggressive esophagectomy has yet to be established. Up to date, the efficacy of adjuvant chemotherapy, radiotherapy, and conventional immunotherapy seems to be disappointed. Surgery might be the most effective treatment for isolated metastasis from melanoma, especially for metachronous disease, although the prognosis remains unsatisfactory.^[3]^ A follow-up study of PMME patients after esophagectomy reveals 70% recurrences and 50% deaths; additionally, all the patients with lymph node metastasis have relapsed within 1 year, which shows that esophagectomy might benefit PMME patients without lymph node involvement.^[4]^ Another study indicates that surgical resection probably is the first choice for PMME without distal metastases.^[5]^

Nevertheless, the clinical benefit of single-stage resection of primary and metastatic melanoma followed by interferon alpha for advanced PMME patients is uncertain, because the reports involving prolonged survival are truly insufficient. Herein, a rare long-term survivor with PMME and localized, resectable pulmonary metastasis is presented, followed by critical review of literatures in terms of the diagnosis, staging, and updated treatment options of this devastating disease.

## 2 Case presentation

A 63-year-old male patient without smoking or drinking history was admitted on June 11, 2014. His major complaints were gradually aggravated dysphagia and fatigue, on suspicion of obstructive disease in upper digestive tract. He had been an athlete before, and then retired in good physical status before admission. His family and social history indicated nothing abnormal. Thorough physical examination of his skin, oral mucosa, eyes, and genital areas failed to identify any superficial lesions. Additionally, laboratory tests including hepatic function, renal function, and serum tumor markers such as carcinoembryonic antigen, cytokeratin 19 fragment, squamous cell carcinoma, neuron-specific enolase, and carbohydrate antigen 125 were all in normal range. Therefore, further endoscopic and radiological examinations were carried out for accurate diagnosis.

**Figure 1:**
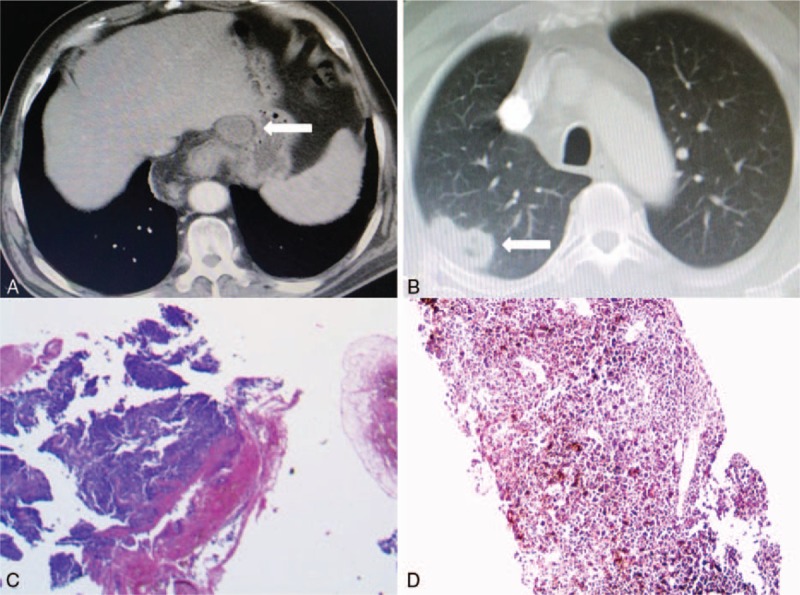
(A) Computed tomography (CT) scan on admission showed a tumor measuring 5.5 cm × 3.5 cm × 3.0 cm in the lower esophagus with enlarged celiac lymph nodes (straight arrow). (B) The concurrent pulmonary lesion of 2.0 cm × 1.0 cm in size located in right upper lobe, (C, D) Postoperative histopathology revealed esophageal and pulmonary melanoma, by H&E staining (×100).

Endoscopic examination revealed a slightly pigmented, irregular mass, which was located in lower esophagus, measuring 5.0 cm × 3.0 cm in size. Fine needle biopsy of the lesion revealed esophageal melanoma, which was confirmed by histopathology. Besides chest and abdomen computed tomography (CT), enhanced cranial magnetic resonance image (MRI) and bone emission computed tomography (ECT) showed enlarged mediastinal, nd also celiac lymph nodes (Fig. 1A), without obvious involvement of supraclavicular lymph nodes. Concurrently, the CT showed an isolated, irregular pulmonary tumor (Fig. 1B). Positron emission tomography was not carried out, because it was not covered by health insurance of this patient.

Therefore, this patient was clinically staged as cT3NxM1 according to the 7th edition of American Joint Committee on Cancer TNM staging system for esophageal cancer. CT-guided percutaneous pulmonary biopsy was avoided, with the aim to diminish the risk of tumor dissemination. Single-stage resection of the esophageal and pulmonary lesions was assumed to be reasonable after multidisciplinary consultation, which was approved by Ethical Committee of Xuzhou Central Hospital. Because the prognosis of this patient probably was extremely poor without targeted antibodies, which he could not afford for financial reasons. After his informed consent, simultaneous Ivor-Lewis esophagectomy and right upper lobectomy were performed successfully, under general anesthesia, after double-lumen endotracheal intubation, followed by systemic dissection of lymph nodes located in mediastinum and abdomen, in accordance with the principles of oncological surgery. The operation time was 290 minutes, without obvious bleeding during the surgery.

Postoperative pathological staining of the specimen revealed pleomorphic cells and abundant melanin granules (Fig. 1C), whereas immunohistochemical tests demonstrated positive expression of human melanoma black 45 (HMB45), microtubule-associated protein tau 1 (MAPT1), melan A and S100, and negative expression of desmin, synaptophysin, and epithelial membrane antigen (EMA), which was consistent with melanoma. The resection margin and dissected lymph nodes were pathologically tumor-negative. In addition, molecular study of the patient indicated mutation of V-raf murine sarcoma viral oncogene homolog B1 (BRAF) V600E.

**Figure 2:**
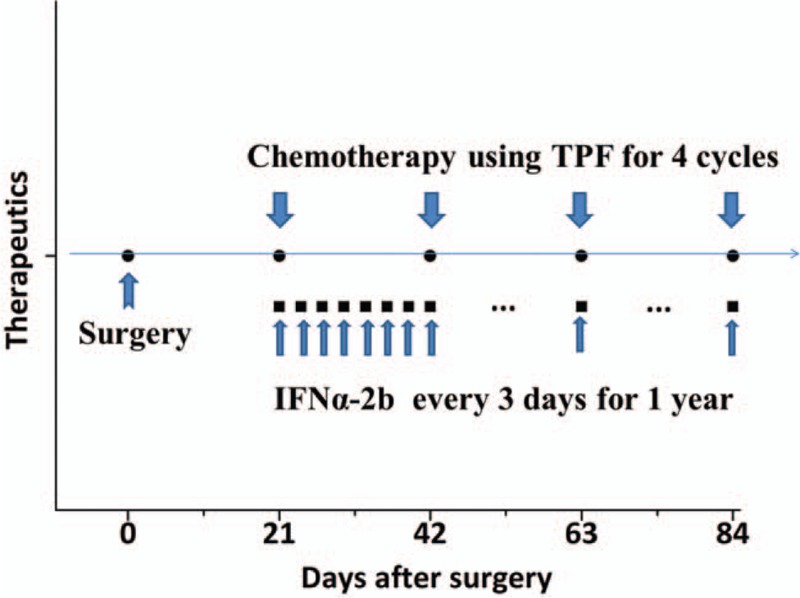
The schematic illustration of therapeutic regimen of the patient.

Based on these findings, a diagnosis of advanced PMME was tentatively established as stage IV (pT3N0M1), because there was insufficient evidence to distinguish synchronous primary pulmonary melanoma from metastasis for this patient. The postoperative recovery was mainly uneventful, and the patient was discharged 14 days after surgery. Subsequently, 4 cycles of adjuvant conventional chemotherapy with an interval of 3 weeks were completed, with controlled moderate toxic effects including thrombocytopenia, leukopenia, nausea, vomiting, and diarrhea. The detailed chemotherapy regimen is as follows: paclitaxel liposome for injection on day 1 and day 8 (135 mg per square meter of body surface area; Nanjing Luye Sike Pharmaceutical Co., Ltd., Jiangsu, China.), tegafur injection on day 2 to 4 (1000 mg per square meter of body-surface area; Shandong Qilu Pharmaceutical Co., Ltd., Jinan, China.) plus cis-platinum on day 2 to 3 (75 mg per square meter of body-surface area; Shandong Qilu Pharmaceutical Co., Ltd., Jinan, China). This patient suffered from moderate leukopenia/ myelosuppression after the second cycle of TPF chemotherapy, and he recovered quickly after the administration of granulocyte colony-stimulating factor (G-CSF). Concurrently, recombinant human interferon alpha-2b (Harbin pharmaceutical group biological engineering Co., Ltd, Harbin, China) was administrated via hypodermic injection thereafter (6000 units every 3 days; Fig. 2), lasting for 1 year.

**Figure 3:**
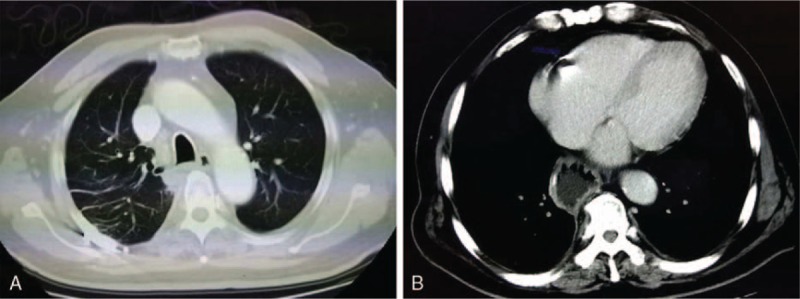
Computed tomography (CT) images excluded recurrence or metastasis 2 years after surgery.

The patient was followed up continuously after the surgery. Chest and abdomen CT, cranial MRI, bone ECT, and thorough physical examination were carried out every 3 months. Encouragingly, the patient survived without loco-regional recurrence or remote metastasis during the follow-up of two and a half years up to now (Fig. 3).

## 3 Discussion

Advanced melanoma is mainly incurable with expensive therapeutics. However, physicians’ empirical misdiagnosis regarding suspicious lesions contributes to delay in treatment of melanoma.^[6]^ Precise diagnosis and timely resection are essential for PMME patients; accordingly, there are several issues that need to be further elucidated, which might contribute to the long-term, recurrence-free survival of this patient.

Firstly, melanoma can present as pink or red instead of pigmented lesion, which could be empirically misdiagnosed as benign or other malignancies.^[7]^ The incidence of distant metastasis form PMME at the initial diagnosis is around 40% to 80%.^[8]^ It is reported that the 1-year and 5-year survival rates of these patients are 51% and 10%, respectively.^[9]^ PMME could be identified by in situ melanoma, radial growth phase, and mixed epithelioid and spindle cell morphology.^[10]^ The typical characteristics of PMME are lobular and pigmented; additionally, intramural metastases, melanocytosis, and melanoma in situ are commonly coexisted.^[8]^ Specifically, PMME should not be excluded directly when the melanin granules are absent. A definite diagnosis could be obtained by positive staining of HMB45, melan A, S100, and neuron-specific enolase.^[8]^ As for the presenting case, the possibility of a regressed or unidentified extra-esophageal melanoma cannot be absolutely excluded.

Secondly, a single-stage, radical resection of the lesion remains preferential for early-stage PMME. Aggressive and systemic lymph node dissection is potentially beneficial for survival of the patients, as lymph node metastasis is an independent prognostic factor.^[11]^ As demonstrated in this case, for selected patients with advanced PMME and isolated remote metastasis, single-stage resection of the localized lesions could be beneficial.

Thirdly, spontaneous regression can be identified histologically in over 25% of primary cutaneous melanomas, and robust immune response could result in effective eradication of tumor cells^[12]^; therefore, conventional immunotherapy after surgery is reasonable for selected patients. High-dose interferon-α2b (IFN-α-2b) is considered to be the mainstay in high-risk melanoma after definitive surgery.^[13]^ Administration of IFN-α-2b for 1 year is associated with improved survival for resected stage IIB to IIIC malignant melanoma.^[14,15]^ Besides, targeted immunotherapy using monoclonal antibody combined with high-dose interleukin-2 (IL-2) is considered to be first-line option for stage IV melanoma.^[16]^ The other therapeutics include intratumoral injection of IL-2, newly emerging anti-programmed cell death 1 receptor (PD-1) agents, anti-programmed death-ligand 1 (PD-L1) agents, and oncolytic vaccines.^[17]^ A meta-analysis of present trials shows that PD-1 and PD-L1 inhibitors are associated with improved response rates and durability, and also tolerable toxicity in advanced melanoma patients.^[18]^

Moreover, immune checkpoint inhibitors are capable of unleashing dormant or exhausted antitumor immunity, which leads to durable responses in melanoma patients.^[19]^ BRAF inhibitors, as compared with conventional chemotherapy, improve progression-free survival of patients with BRAF mutation, which is found in 50% of the advanced melanoma patients.^[20]^ BRAF mutation, an independent prognostic factor of resected stage IIIB and IIIC melanoma, is associated with rapid progression and loco-regional recurrence of the disease.^[21]^ Similarly, BRAF-targeted therapy augments the immune response of the host, which is down-modulated before resistance occurs.^[22]^ Nevertheless, the efficacy of BRAF inhibitors is limited and transient by the emergence of acquired drug resistance.^[23]^ A combination of BRAF and mitogen-activated protein kinase/extracellular signal-regulated kinase activator kinase (MEK) inhibitors could be considered as first-line or second-line therapy for BRAF mutant, previously untreated melanoma patients, as compared with BRAF inhibitor alone, which might reverse or delay the emergence of acquired resistance.^[24,25]^ Moreover, triple combination of immunotherapy, BRAF and MEK inhibitors may be another choice for refractory BRAF(V600E) mutant patients with metastatic melanoma.

It is noteworthy that the potential reasons regarding the efficacy of radical surgery, chemotherapy, and immunotherapy regimen for this patient is just theoretical, including good immune status of the patient, complete resection of the lesions, and a rational combination of local and systemic treatment. Furthermore, the evidence level of single case report is actually low.

## 4 Conclusion

In summary, timely complete resection of the lesions, followed by concurrent immunotherapy and chemotherapy, might be a reasonable choice for strictly selected PMME patients with isolated/ localized, resectable metastasis. However, high-quality, large-scale studies with long-term follow-up are truly needed to further evaluate the multidisciplinary therapeutic regimens for PMME patients, and to elucidate the detailed inclusion/ exclusion criteria of aggressive resection.
